# Cell penetration efficiency analysis of different atomic force microscopy nanoneedles into living cells

**DOI:** 10.1038/s41598-021-87319-3

**Published:** 2021-04-08

**Authors:** Marcos Penedo, Tetsuya Shirokawa, Mohammad Shahidul Alam, Keisuke Miyazawa, Takehiko Ichikawa, Naoko Okano, Hirotoshi Furusho, Chikashi Nakamura, Takeshi Fukuma

**Affiliations:** 1 Nano Life Science Institute (WPI-NanoLSI), Kanazawa University, Kanazawa, 920-1192 Japan; 2 Division of Electrical Engineering and Computer Science, Kanazawa University, Kakuma-Machi, Kanazawa, 920-1192 Japan; 3 Division of Nano Life Science, Kanazawa University, Kakuma-Machi, Kanazawa, 920-1192 Japan; 4 Faculty of Frontier Engineering, Kanazawa University, Kakuma-Machi, Kanazawa, 920-1192 Japan; 5 AIST-INDIA Diverse Assets and Applications International Laboratory (DAILAB), Cellular and Molecular Biotechnology Research Institute (CMB), National Institute of Advanced Industrial Science and Technology (AIST), Tsukuba, Ibaraki 305-8565 Japan; 6 Present Address: Bioengineering department, Ecole Polytechnique Fédérale de Lausanne, EPFL STI IBI-STI LBNI, Lausanne, Switzerland

**Keywords:** Nanoscale biophysics, Atomic force microscopy, Scanning probe microscopy, Applications of AFM, Biosensors, Nanostructures, Atomic force microscopy, Scanning probe microscopy

## Abstract

Over the last decade, nanoneedle-based systems have demonstrated to be extremely useful in cell biology. They can be used as nanotools for drug delivery, biosensing or biomolecular recognition inside cells; or they can be employed to select and sort in parallel a large number of living cells. When using these nanoprobes, the most important requirement is to minimize the cell damage, reducing the forces and indentation lengths needed to penetrate the cell membrane. This is normally achieved by reducing the diameter of the nanoneedles. However, several studies have shown that nanoneedles with a flat tip display lower penetration forces and indentation lengths. In this work, we have tested different nanoneedle shapes and diameters to reduce the force and the indentation length needed to penetrate the cell membrane, demonstrating that ultra-thin and sharp nanoprobes can further reduce them, consequently minimizing the cell damage.

## Introduction

For the last years, the atomic force microscope (AFM) has become a powerful tool for studying biological systems, mainly for its capability to measure in liquids with nanoscale resolution. Measuring tissues, cells, proteins or smaller biological molecules in their physiological conditions gives us access to valuable information about their real ‘in vivo’ structure, dynamics and functionality, leading towards disruptive medical and biological applications.

However, the range of possible applications of the AFM system is not restricted to topographical related measurements. The cantilever tip can be used as a nanoprobe sensor or in drug delivery systems. For instance, the tip of a commercial AFM cantilever can be introduced into living cells to extract mRNA, to be analyzed afterwards with polymerase chain reaction (PCR) tests^1,2^. The AFM tip is inserted into the cytoplasm to collect mRNA and placed after into PCR tubes. By applying this method, it is possible to perform quantitative measurements of mRNA at different loci within individual living cells. Nevertheless, the use of commercial cantilever tips as purchased could damage the cells due to the large size of the typical pyramidal or conical tips, in the order of several micrometers. To overcome this problem and thus minimize the cell damage, tip size reduction and special AFM probe designs are required. With this in mind, other studies^3^ monitored mRNA expression in a single living cell by using an atomic force microscope equipped with a tailored nanoprobe^4,5^, immobilizing a biotin-modified molecular beacon onto an ultrathin needle via neutravidin to detect glyceraldehyde-3-phosphate dehydrogenase (GAPDH) mRNA inside living cells. The reduced needle (several micrometers long and 200–400 nm of diameter) penetrates the cells producing a minimum damage on their structure. Moreover, surface functionalized nanoprobes can also be used for the detection of other molecules such as cytoskeletal proteins, intermediate filaments^6^, actin microfilaments^7^ or microtubules^8^ without any cell labeling, being also possible to control gene expression^9^. Furthermore, the nanoprobe can be used for molecular delivery, immobilizing the target molecule at the surface of the nanoneedle, which detaches from the needle surface once the nanoprobe is inside the living cell^10–12^.

But the use of nanoneedles for molecular detection or delivery inside the cells is not always based on AFM cantilever tips. Cell membranes mechanical disruption for cytosolic molecular delivery through arrays of nanoneedles^13–18^ has demonstrated intracellular delivery capabilities without causing serious damage to cells, where the molecular delivery can be performed in parallel for thousands of cells, increasing the delivery efficiency. These nanoneedle arrays can also be used for cell separation, which allows the simultaneous insertion of multiple needles into multiple cells for transferring them on a new substrate in parallel^19^.

The most important aspect in all these cell membrane mechanical disruption related techniques (with or without using AFM cantilevers based nanoprobes) is to improve the efficiency of the nanoneedle insertion, maximizing the cell penetration probability while minimizing the cell damage by reducing the nanoprobe size. Several experimental studies have shown that flat nanoneedles lead to higher penetrations probabilities, lower penetration forces, and shorter indentation lengths^4,5,20^ compared to conical nanoneedles. This reduces cell damage and enhances penetration rates since large indentation depth relative to the height of the cell may cause serious damage, and/or undesirable mechanotransduction. The authors suggested that this is due to the shear stress produced by the tip’s contact area on the cell membrane, which is higher in flat than in cone-shaped or pyramidal tips, increasing the penetration efficiency and reducing the required force to pierce cells. However, these experiments were never performed using ultrathin nanoneedles presenting diameters on the range of several tens of nanometers, which could dramatically decrease the penetration force and indentation length compared to flat tips.

Besides the experimental studies cited above about how to improve penetration efficiency and reduce cell membrane damage during penetration, great theoretical effort has been performed too. Some works have shown that a reduced indentation speed lowers penetration forces in spherical^21^ and conical tips^22^ in lipid membranes. Also, simulations affirm that penetration forces decrease when the curvature of a nano-cone tip increases, and large nano-tip radius displays a high penetration force in the indentation process^22^. In addition, theoretical analysis of surface functionalization on nanoprobes suggests that tailored hydrophobic and hydrophilic patterns decrease the penetration force and cell damage^22–25^, but the complicated microfabrication process required hinders this approach.

In order to verify if reducing the diameter of the nanoprobes to several tens of nanometers would further decrease the required penetration force and indentation length, we have microfabricated long and ultra-thin nanoneedles with the focused ion beam (FIB), performing a study on HeLa cells where, in a systematic analysis, we have compared flat and sharp tips with different diameters.

## Results and discussion

**Figure 1:**
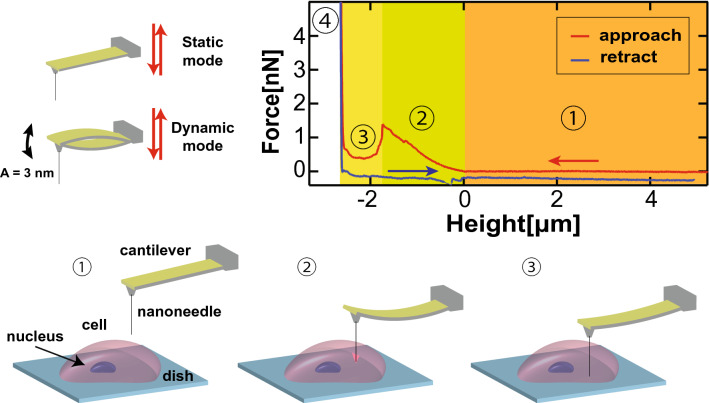
Schematic of the cell penetration experiments, where the nanoneedle above the cell is vertically moved down until a specific force or oscillation amplitude set point is reached, retracting afterwards. The nanoprobe can be oscillated a few nanometers in amplitude during the process (dynamic mode) or kept static (static mode).

Aimed at the abovementioned theoretical analysis that reduced conical tips would reduce the forces required to penetrate lipid membranes, we have studied the required penetration force (F_P_) and indentation length (I_L_) on HeLa cells using ultra-thin nanoprobes, comparing these nanoneedles with flat and thicker ones. Sharp nanoprobes will also reduce the required force to overcome the cortical actin scaffold underneath the cell membrane, which has as well an important contribution to the force needed to pierce the cell. For the sake of space, although along the text we will only refer to cell membrane penetration, the nanoprobe will only enter the cell when it pierces both cell membrane and its cortical actin. Figure 1 depicts a schematic of the cell penetration experiments. FIB microfabricated nanoneedles on commercial cantilevers (OPUS 3XC-GG, spring constant 0.3 N/m) were inserted into living cells apart from the nucleus at a speed of 10 µm/s, recording the cantilever deflection during the process, until a specific set-point is reached, withdrawing the nanoprobe afterwards at the same speed. In the case of the static mode, where the cantilever is not simultaneously oscillated, the cantilever vertical force is used for the set point (5 nN). When the nanoprobe is far from the cell, a zero-value cantilever deflection is recorded, corresponding to the area 1 in the bottom graph of Fig. 1. Once the cantilever is in contact with the cell membrane and is vertically moved further down, it starts to bend, increasing the deflection and the vertical force accordingly, as shown in the area 2 in Fig. 1. Eventually, the nanoneedle penetrates through the cell membrane, stopping the increase of the cantilever bending, as depicted in the area 3. Finally, the nanoprobe reaches the dish surface, which leads to a sharp increase of the cantilever bending, as depicted in the area 4. Apart from this static mode for the cell penetration experiments, we have repeated the same experiments with the same nanoneedles, but also vibrating the cantilevers while performing the force versus distance (F_z_) curves, at their second resonance mode with an amplitude of around 3 nm, investigating if oscillating the cantilever while penetrating the cell has an effect on the F_P_ and I_L_. Being able to oscillate the cantilever while penetrating the cell would also provide more information channels in the amplitude and phase signals during the F_z_ curves. As in the static mode, in the dynamic mode the nanoneedle is vertically moved down until the cantilever oscillation amplitude is decreased by 60%, retracting the nanoprobe after. As the cantilevers used in the experiments presented low resonance frequencies due to their low spring constant, we have used the second resonance mode to avoid the high noise level and the spurious resonances around the first resonance mode, produced by the cantilever’s acoustic excitation. More detailed information about nanoprobes microfabrication, cell culturing and sample preparation can be found in the “Materials and methods” section.

**Figure 2:**
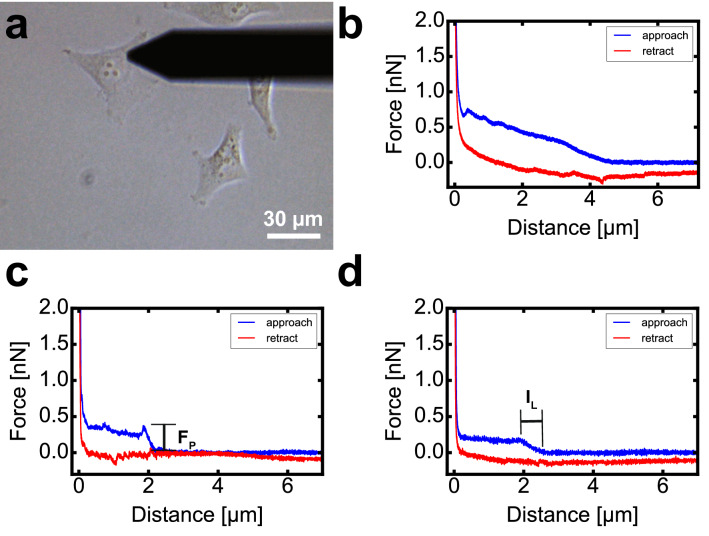
(**a**) Optical view of the cantilever with the nanoprobe above a HeLa cell during a cell penetration experiment. Examples of force versus distance curves, where the nanoneedle did not penetrate the cell (**b**), and where the nanoprobe was successfully inserted into the cell (**c**,**d**). We consider a cell penetration to be successful when either the cantilever’s force drastically drops (**c**) or the force presents a long plateau after the nanoprobe contacts the cell surface (**d**).

With the help of an optical microscopy, we placed the nanoprobe located at the end of the cantilever at the desired position on the cell, at some distance from the nucleus, as shown in the example of the Fig. 2a. Then, a F_z_ curve is performed, recording the vertical force during the approach and retract segments. We only performed one F_z_ curve in each cell to prevent artifacts due to cell changes after its penetration, recording first around 50 F_z_ curves on the static mode and after around other 50 F_z_ curves on the dynamic mode, always using the same nanoprobe to compare both static and dynamic modes. After finishing the experiments with every tested nanoprobe, a scanning electron microscopy (SEM) image was taken to check if the nanoprobe was still intact. Figure 2b–d shows examples of the performed Fz curves on HeLa cells. Figure 2b displays the typical behavior when the cell penetration does not happen, showing a continuous increment of the cantilever’s vertical force until it reaches the surface, where a sharp increase follows. On the contrary, when the nanoneedle penetrates the cell membrane, the vertical force stops increasing, displaying two different behaviors after cell membrane perforation: either the cantilever vertical force drastically drops (Fig. 2c), or the force presents a long plateau (Fig. 2d). We attribute the difference to how the needle’s tip goes through the membrane, smoother when the Fz curves do not present a sharp drop. Furthermore, these smoother membrane penetrations as in Fig. 2d also display smoother curves while inside the cell, with no other small peaks on the curve as in Fig. 2c, possible due to lower friction between the nanoneedle walls and the cell membrane. In addition, when the nanoprobe perforates the cell membrane, the retract segment of the F_z_ displays rather a plateau behavior (Fig. 2c-d) than a more elastic curve-shaped behavior (Fig. 2c). In the case of a successful penetration, F_P_ and I_L_ were calculated, along with all the related statistical values (average, median, quartiles, maximum and minimum). We define F_P_ as the difference in the cantilever vertical force between the contact point, when the nanoneedle contacts the cell membrane, and the position when the actual membrane penetration happens, sharply dropping (Fig. 2c) or stopping the raise (Fig. 2d) of the vertical force. The I_L_ is the vertical distance between contact and penetration points. We determine the contact point in the F_z_ curves where the cantilever vertical deflection starts to increase from its vertical deflection baseline; and the penetration point where either the cantilever vertical deflection sharply drops, or the monotonously rising vertical force drastically stops increasing and stabilizes forming a plateau, which produces an acutely change on the F_z_ curve slope (further information about the interpretation of the F_z_ curves and a confocal image of the nanoprobe inside a living cell can be found in the Supplementary Information).

**Figure 3:**
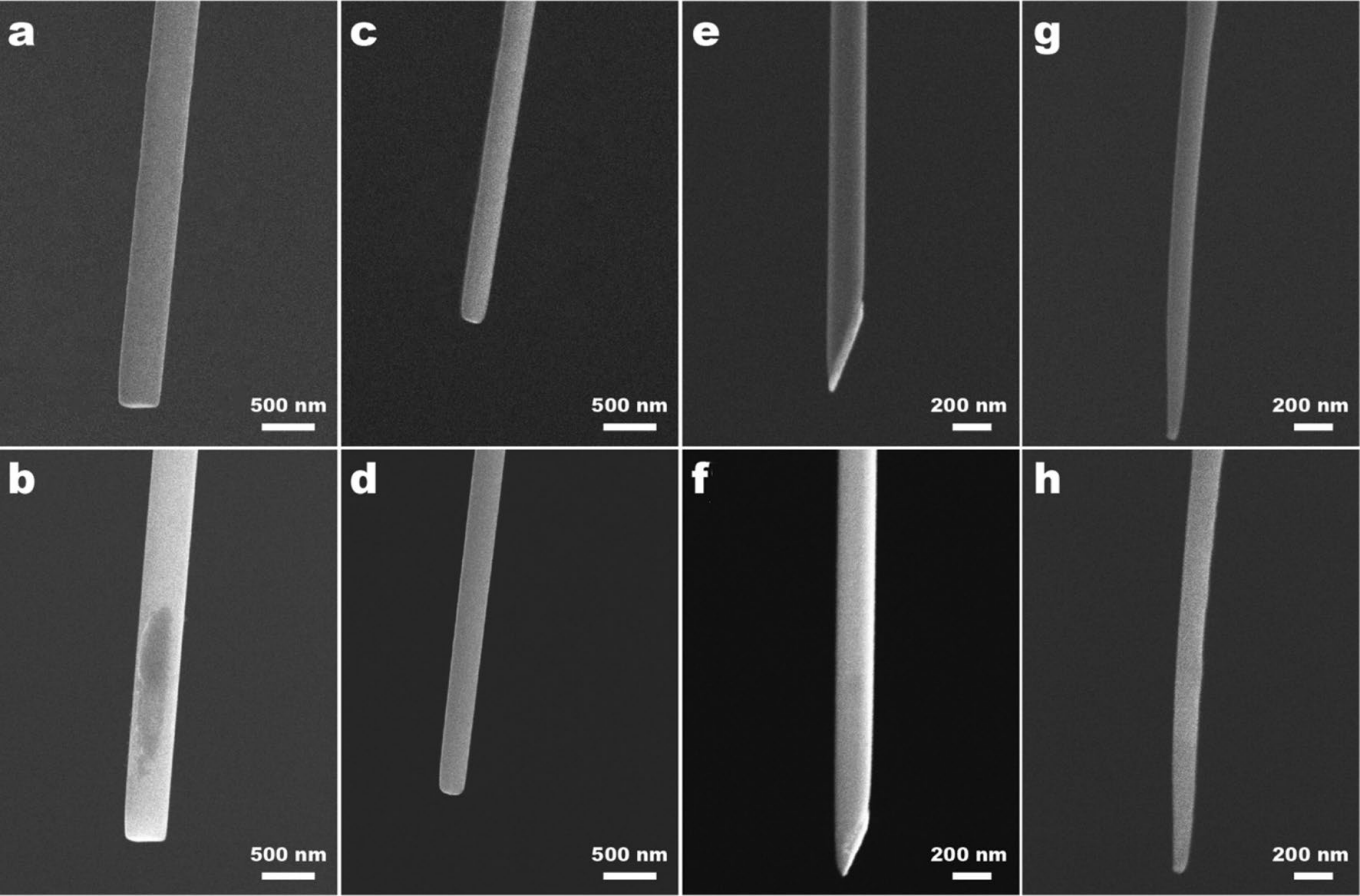
SEM images of the different nanoneedles used, before (**a**,**c**,**e**,**g**) and after (**b**,**d**,**f**,**h**) the cell penetration experiments: 400 nm flat (**a**,**b**); 200 nm flat (**c**,**d**); 200 nm sharp (**e**,**f**); and sharp (**g**,**h**).

Figure 3 displays SEM images of the nanoprobes before (top row) and after (bottom row) the penetration experiments. We have used four different types of nanoprobes. Two of them present a flat end, featuring diameters of 400 nm (Fig. 3a,b) and 200 nm (Fig. 3c,d). A third one has a sharp end, with a diameter of 200 nm (Fig. 3e,f). And finally, a sharp conical nanoneedle, with a diameter around 100 nm at 2 µm from the tip (Fig. 3g,h). Do note that further decreasing of the conical nanoneedle diameter would lead to a collapse and bending of the nanoprobe (more detailed information about the structural damage of the FIB milling nanoprobes is shown in the Supplementary Information). It is also worth mentioning that we did not appreciate changes on the shape of the nanoneedles after the measurements, preserving the same sharpness or flatness, exhibiting thus high robustness and mechanical stability, including the sharp nanoprobe.

**Figure 4:**
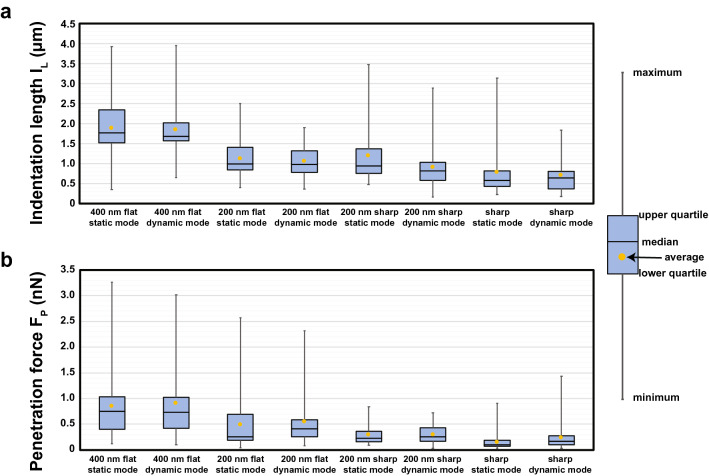
Results of the cell penetration experiments, comparing the indentation length (I_L_) and penetration force (F_P_) for the different type of nanoprobes and penetration methods, showing for each case the average, median, upper and lower quartile, and the data interval range.

An overview of the experiments results can be found in Fig. 4, with the statistical values summarized in the Table 1. As expected, the 400 nm flat nanoprobe presents much lower penetration success (70% in static mode, 50% in dynamic mode) compared to the 200 nm flat (94% in static mode, 94% in dynamic mode), as well as higher F_P_ and I_L_, for both static and dynamic modes. The large contact area of the 400 nm flat nanoprobes hampers the membrane penetration, compressing the cell rather than penetrating it, whose effect impacts in the long indentation length required for penetration, around 1800 nm in both modes. If we compare the results of the 200 nm flat nanoprobe in our experiments in static mode with previous studies in the literature, the results are similar. In our case, the penetration probability and the average F_P_ and I_L_ are 94%, 0.49 nN and 990 nm, respectively; compared to 92%, 0.65 nN and 610 nm of previous studies ^5^. On the other hand, our experiments with the 200 nm sharp nanoprobe in the static mode display slightly higher penetration success (98%) and average I_L_ = 1190 nm, and lower values in the average F_P_ = 0.29 nN, compared to the 200 nm flat. These results present some discrepancies with the work of Obataya et al., where they also tested 200 nm sharp nanoprobes. In that study, the average F_P_ and I_L_ were 70%, 0.78 nN and 1200 nm, respectively^5^. We attribute this discrepancy to our capacity to mill sharper tips in our system, where the tip end is sharper than the contour of the flat end of their flat nanoprobes, which facilitates the membrane rupture and its subsequent perforation.

**Table 1:** Statistical results of the penetration experiments, displaying for each nanoprobe type and penetration method the penetration probability, and the average and median of the penetration force (F_P_) and indentation length (I_L_).

Nanoneedle type	Probability [%] (success/total)	Average F_P_ (nN)	Median F_P_ (nN)	Average I_L_ (nm)	Median I_L_ (nm)
400 nm flat, static mode	70 (35/50)	0.85 ± 0.61	0.75	1880 ± 730	1770
400 nm flat, dynamic mode	50 (25/50)	0.91 ± 0.73	0.73	1840 ± 650	1680
200 nm flat, static mode	94 (47/50)	0.49 ± 0.51	0.26	1120 ± 460	990
200 nm flat, dynamic mode	96 (48/50)	0.55 ± 0.48	0.41	1050 ± 380	980
200 nm sharp, static mode	98 (50/51)	0.29 ± 0.19	0.23	1190 ± 670	945
200 nm sharp, dynamic mode	98 (49/50)	0.29 ± 0.17	0.26	900 ± 550	810
Sharp, static mode	98 (48/49)	0.15 ± 0.15	0.10	770 ± 600	575
Sharp, dynamic mode	98 (50/51)	0.23 ± 0.24	0.17	700 ± 410	645

We found that if the nanoprobes are milled thinner, F_P_ and I_L_ further decrease. We were able to decrease the diameter of the nanoneedles to several tens of nanometers. Below this value the nanoneedle starts to collapse and bends, excluding its use for cell penetration experiments. In this study, we have used a sharp nanoprobe presenting around 100 nm in diameter 2 µm away from the tip end, as depicted in Fig. 3g,h. As shown in the Table 1, penetration experiments in static mode decreases F_P_ by a factor of two (0.15 nN), decreasing I_L_ as well to a value of 770 nm. It should be noted that F_P_ and I_L_ values are more concentrated around their average value, since the range between the lower and upper quartiles is also smaller, showing less dispersion on the points around the average measured property, as shown in Fig. 4. This clearly shows that further sharpening the nanoprobe by decreasing the nanoneedle’s diameter dramatically decreases F_P_, improving the I_L_ at the same time. This is important since lower F_P_ and I_L_ will reduce cell damage and the undesirable mechanotransduction in experiments where a nanoneedle must be inserted into a cell, increasing the survival rate of the cells. As for the penetration probability, the sharp nanoprobes also keep a high value of 98%. F_z_ curves of the used nanoprobes performed on the Petri dish surface as a control experiment are shown in the Supplementary Information.

Regarding the results in the case of the F_z_ curves in dynamic mode, we do not appreciate large differences, which are always on the range of the experimental error, as shown in Fig. 4 and Table 1. This means that it is possible to oscillate the cantilever while penetrating the cells, without compromising its penetration performance. This opens the possibility to use the cantilever’s amplitude and phase while performing cell penetration experiments in bio-sensing or molecular recognition experiments, which provides new measurables (amplitude and phase signals) as extra information channels, and increases the signal to noise ratio due to the high frequencies used to excite microcantilevers and the utilization of lock-in amplifiers.

## Conclusions

Different nanoneedles were fabricated from silicon AFM tips by FIB milling processes, presenting different diameters and tip shapes. In the case of static mode penetration experiments, lower F_P_ and I_L_ values were achieved with sharper nanoprobes, compared to the flat ones. Furthermore, decreasing the diameter of the nanoprobes also reduces F_P_ and I_L_, which is important since lower F_P_ and I_L_ will reduce cell damage and the undesirable mechanotransduction, increasing the survival rate of the cells. Penetration dynamic modes do not display large differences, paving the way to utilize the cantilever’s amplitude and phase in bio-sensing or molecular recognition experiments, since they provide extra information channels, increasing the signal to noise ratio at the same time. In the future, new micro-fabrication techniques and materials should be tested in order to further reduce the nanoneedle dimensions, to minimize F_P_ and I_L_. These results will help improve all the techniques where a thin nanoneedle is required to be inserted into a living cell in both based and non-based AFM systems, such as bio-sensing, molecular recognition, drug delivery or cell separation.

## Materials and methods

### Cell culture

HeLa cells (Cell Resource Center for Biomedical Research, Institute of Development, Aging and Cancer, Tohoku University) were cultured in Dulbecco's Modified Eagle's Medium (DMEM, Fujifilm Wako Pure Chemical Corporation), supplemented with 10% FBS (fetal bovine serum, Biosera) and 1% PS solution (penicillin-streptomycin, Fujifilm Wako Pure Chemical Corporation). The day before the cell penetration experiments, cells were harvested from a Petri dish with 0.05% trypsin/EDTA for 2 min at 37 °C and centrifuged at 1400 rpm for 3 min. Then, cells were seeded onto a 35 mm plastic cell culture dish (TPP, Techno Plastic Products AG, Trasadingen, Switzerland), and cultured with DMEM for 24 h. Prior to the measurements, the cell culture medium was replaced with CO_2_-independent Leibovitz L-15 medium (Fujifilm Wako Pure Chemical Corporation), supplemented with 5% penicillin-streptomycin solution.

### AFM nanoneedles microfabrication

The long AFM microcantilever (500 µm length, 0.3 N/m spring constant) of an OPUS 3XC-GG cantilever chip (MikroMasch) was used to microfabricate the nanoprobes. Their silicon tetrahedral tip was milled by the focused ion beam (FIB) technique in a Helios G4 CX Dual Beam system (FEI, Thermo Fisher Scientific). The cantilever tip was sequentially milled alternating its front and lateral sides, reducing its diameter gradually until the desired value. The accelerating voltage was kept at 16 kV at a working distance of 4 mm, reducing the current gradually from 1.3 nA to 15 pA while the nanoneedle’s diameter reduces to avoid tip damage, and to increase milling resolution. The nanoneedles were FIB microfabricated with an angle of 10 degrees between their long axis and the cantilever plane to correct the mounting angle on the AFM cantilever holder. Prior to the experiments, the nanoneedles were introduced in a soft plasma etching device for 10 min at 2 mA (Meiwafosis, Japan) to remove any contaminant on the nanoprobe surface. Plasma etching increases also the hydrophilicity of the nanoprobe surface, which may affect therefore the experiments results. However, as we have used the same preparation protocol for all the nanoprobes to ensure they presented the same surface properties, we were able to compare the results between the different nanoprobes (sharp and flat ones), avoiding thus differences on the F_z_ curves originated from differences in the surface chemistry.

### AFM cell penetration experiments

Cell penetration measurements were performed in a JPK Nanowizard 4 BioAFM (Bruker Nano GmbH, Berlin, Germany). The nanoneedles were inserted into living cells at a speed of 10 µm/s, recording the cantilever deflection during the process until a specific set-point was reached, withdrawing subsequently the nanoprobe at the same speed. The penetration experiments were carried out in two different ways: (1) the nanoneedle above the cell was vertically moved down until a set point of 5 nN is reached, retracting afterwards (static mode); (2) similarly to the previous mode, the nanoneedle was vertically moved down, but in this case the cantilever was simultaneously vibrated with an oscillation amplitude of around 3 nm at its second resonance mode (40 kHz ~ 60 kHz), until the cantilever oscillation amplitude was decreased by 60%, retracting the nanoprobe after (dynamic mode). The number of penetration experiments was around 50 times per nanoprobe and method, always at some distance from the nucleus to avoid cell damage. Sensitivity and cantilever stiffness were calibrated using the thermal noise method as implemented in the JPK AFM control software. All the AFM experiments were performed at 37 °C in physiological conditions.

### Statistical methods

We consider a cell penetration to be successful when either the cantilever vertical force drastically drops, or the force presents a long plateau after the nanoprobe contacts the cell surface. After finding the cell contact and penetration points in the force versus distance curves, the indentation length (I_L_) and penetration force (F_P_) were calculated. All the statistical analysis was performed on Microsoft Excel, calculating for each nanoprobe and mode the different statistical values for the penetration force and indentation length: penetration success ratio, average, median, minimum, maximum, range, and upper and lower quartile.

## Supplementary Information

Supplementary Information
