# Supplementary material for: Cell penetration efficiency analysis of different atomic force microscopy nanoneedles into living cells
Source: Sci Rep. 2021 Apr 8;11:7756. doi: 10.1038/s41598-021-87319-3 (PMC8032717; doi:10.1038/s41598-021-87319-3)
Supplement: Supplementary file 1 — Supplementary Information [file 41598_2021_87319_MOESM1_ESM.docx]

**Cell penetration efficiency analysis of different atomic force microscopy nanoneedles into living cells**

**Marcos Penedo**1,*,†**, Tetsuya Shirokawa**2**, Mohammad Shahidul Alam**3**, Keisuke Miyazawa**1,2,4**, Takehiko Ichikawa**^1^**, Naoko Okano**^1^**, Hirotoshi Furusho**1**, Chikashi Nakamura**5**, Takeshi Fukuma**1,2,3,4,*

1Nano Life Science Institute (WPI-NanoLSI), Kanazawa University, Kanazawa 920-1192, Japan

2Division of Electrical Engineering and Computer Science, Kanazawa University, Kakuma-machi, Kanazawa 920-1192, Japan

^3^Division of Nano Life Science, Kanazawa University, Kakuma-machi, Kanazawa 920-1192, Japan

^4^Faculty of Frontier Engineering, Kanazawa University, Kakuma-machi, Kanazawa 920-1192, Japan

5AIST-INDIA Diverse Assets and Applications International Laboratory (DAILAB), Cellular and Molecular Biotechnology Research Institute (CMB), National Institute of Advanced Industrial Science and Technology (AIST), Tsukuba, Ibaraki 305-8565, Japan

^†^ Current address: Bioengineering department, Ecole Polytechnique Fédérale de Lausanne, EPFL STI IBI-STI LBNI, Lausanne, Switzerland

[* fukuma@staff.kanazawa-u.ac.jp](mailto:*%20fukuma@staff.kanazawa-u.ac.jp), [marcos.penedo@epfl.ch](mailto:marcos.penedo@epfl.ch)

**
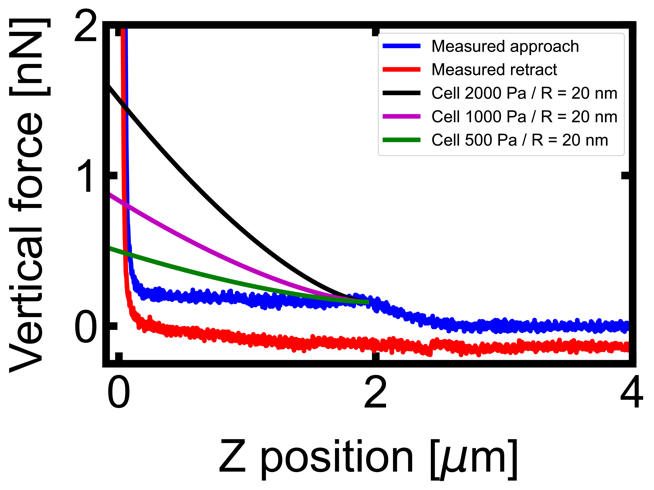
Interpretation of the F_z_ curves displaying smooth penetration**

**Figure S1**. **Experimental and simulated cell penetration F_Z_ curves**.

Measured (blue and red curves) and theoretically calculated (back, violet and green curves) F_z_ curves. The measured F_z_ curve present the typical behavior of a smooth cell penetration with a plateau after the nanoprobe overcomes the membrane and its cortex. In contrast to the flat behavior of the vertical force after the cell is pierced, the theoretically calculated F_z_ curves display the continuous rising of the vertical force expected for the indentation force in the Hertz model used for the theoretical calculations. This plateau cannot be originated by pushing the cell membrane inside the cell after overcoming the actin cortex. In that case, a monotonous increase of the force would be also expected, as reported several studies in membrane mechanics of suspended lipid bilayers [1-3], which can be considered analogous to an aperture/pore on the cortex with a suspended cell membrane. Furthermore, the cell membrane is attached to the cortex through membrane proteins, hindering the membrane pushing inside the cell since it has to slide over the actin cortex (unless the whole cortex is being pushing down, increasing hence the vertical deflection as well), which should lead to a force increase on the cantilever. Figure S1 shows some examples of theoretically calculated F_z_ curves using the Hertz contact mechanics model, where even considering extremely low equivalent stiffness (as low as 500 Pa) of the cell membrane, a noticeable monotonous rising vertical force would be expected. In the measured curves, the plateau does not show a vertical force rising, a clear indication that the nanoprobe entered the cytosol. There is some friction between the nanoprobe and the cell, which explains the force offset of the plateau, and why the force is not decreasing to zero after penetration.

**Figure S2**. **a) Confocal image of a cross section of the cell with the nanoprobe inside, after recorded the force versus distance approach curve displayed in b)**.

The penetration was also confirmed by confocal microscopy, where a confocal image was acquired after cell penetration. Once the approach is finished when the nanoprobe reaches the set-point on the dish surface, it is kept there with the feedback on, imaging the cell and the nanoprobe with the confocal microscope. An example is shown in Figure S2a (after having performed the force distance curve depicted on Figure S2b) where the cytosol of the cell was stained, and the nanoprobe can be clearly identified in the black needle-like region inside the cell that does not show any fluorescence. It can be also appreciated that the nanoprobe reached the dish surface, and it does not produce any invagination on the top cell membrane (neither a deformation on the cell), confirming a perforation of the plasma cell membrane.

***Theory and parameters used for the curves in Figure S1***

For the theoretical calculations of the black, violet and green curves we have used Hertz’s contact mechanics

$$F=\frac{4}{3}E^{*}R^{\frac{1}{2}}d^{\frac{3}{2}}$$

where

$$\frac{1}{E^{*}}=\frac{1-\nu_{1}^{2}}{E_{1}}+\frac{1-\nu_{2}^{2}}{E_{2}}$$

And $F$ is the vertical applied force of the tip, $R$ the tip radius, $d$ the tip indentation, ($E_{1},\nu_{1}$) and ($E_{2},\nu_{2}$) are the elastic moduli and Poisson's ratios associated with the tip (1) and cell (2). In all the cases, we have considered a silicon tip ($E_{1} = 169 GPa,\nu_{1}= 0.27$) and the cell as an incompressible material ($\nu_{2}$ = 0.5). In the different calculated curves, we have varied the tip radius and the Young’s modulus of the cell covering typical values of the cell elasticity:

- Black curve: $R$ = 20 nm, $E_{2} = 2000 Pa$
- Violet curve: $R$ = 20 nm, $E_{2} = 1000 Pa$
- Green curve: $R$ = 20 nm, $E_{2} = 500 Pa$

The cantilever spring constant was taken from the calculated value of the OPUS 3XC-GG cantilever used for the measured blue and red curves $k =$0.3 N/m.

***Protocol for cytosol staining and confocal imaging***

HeLa cells (Cell Resource Center for Biomedical Research, Institute of Development, Aging and Cancer, Tohoku University) were cultured in Dulbecco's Modified Eagle's Medium (DMEM, Fujifilm Wako Pure Chemical Corporation), supplemented with 10% FBS (fetal bovine serum, Biosera) and 1% PS solution (penicillin-streptomycin, Fujifilm Wako Pure Chemical Corporation). The day before the experiments, cells were harvested from a Petri dish with 0.05% trypsin/EDTA for 2 minutes at 37 °C and centrifuged at 1400 rpm for 3 minutes. Then, cells were seeded onto a 35 mm glass cell culture dish (Ibidi) and cultured with DMEM for 24 hours. To stain the cytosol, 10 ml of CO_2_-independent Leibovitz L-15 medium (Gibco, (-) Phenol red) was added to 3 µl of calcein-AM stock solution (Dojindo, 1mmol/L). On the day of the experiment, the cell culture medium DMEM was replaced with the 2 ml solution of L-15 and calcein-AM, and kept in the incubator for 15 minutes. Finally, the medium was substituted with 2 ml of L-15 ((-) Phenol red).

Confocal imaging experiments were performed in a STED-confocal microscope (Abberior Instruments) combined with JPK NanoWizard ULTRA Speed 2 (Bruker Nano GmbH, Berlin, Germany). The cells were observed with a 100x oil-immersed objective lens. Images were taken using Imspector software. The excitation laser wavelength was set at 488 nm to produce the confocal images of the stained cytosol, analyzed after by ImageJ software. A sharp FIB milled nanoprobe of an OPUS 3XC-GG cantilever was inserted into the living cell with a speed of 10 µm/s, avoiding the cell nucleus. To acquire the confocal image after cell penetration, the nanoprobe was kept inside the cell in contact with the surface during the confocal image acquisition with the feedback on, retracting the nanoprobe afterwards with a speed of 10 µm/s. It is important to apply a sufficient set-point force to penetrate the cell membrane and reach the substrate.

[1] Mey, I., Stephan, M., Schmitt, E. K., *et al*. Local Membrane Mechanics of Pore-Spanning Bilayers. *Journal of the American Chemical Society* 131, 7031–7039 (2009).

[2] Simon, A., Gounou, C., Tan, S., Tiefenauer, L., Di Berardino, M., ad Brisson, A. R. Free-standing lipid films stabilized by Annexin-A5. *Biochimica et Biophysica Acta (BBA) – Biomembranes* 1828, 2739-2744 (2013).

[3] Janshoff, A., and Steinem, C. Mechanics of lipid bilayers: What do we learn from pore-spanning membranes? *Biochimica et Biophysica Acta (BBA) - Molecular Cell Research* 1853, 2977-2983 (2015).


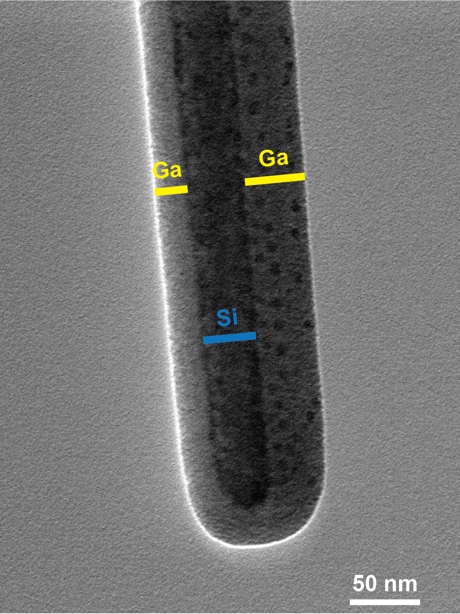
**Gallium (Ga) implantation on FIB milled cantilever tips**

**Figure S3**. **Transmission electron microscopy (TEM) of a FIB milled cantilever tip**.

An example of a needle-like nanoprobe tip fabricated by FIB milling displayed in Figure S3. When attempting to further reduce the nanoprobe diameter and tip radius the needle collapsed as a result of the FIB milling, preventing us from reaching sharper nanoprobes.

The main problem related to the microfabrication of thinner needle-like nanoprobes is the Ga implantation during the milling process, which can reach to 20-30 nm depth (area indicated in yellow in Figure S3). As we fabricate the nanoprobes milling around the original cantilever tip, the implanted area is around the whole tip surface. The Ga implanted volume destroys the crystallinity of the silicon tip, worsen the mechanical properties and leading to the nanoprobe bending. Milling cantilever tips below diameters of 100-150 nm leads to implantation of Ga over the whole nanoprobe (completely removing the silicon crystal depicted in blue in Figure S3), resulting in a bending of the needle due to mechanical damage produce by the Ga implantation. When attempting to further reduce the nanoprobe diameter and tip radius the needle collapsed.

**Force-distance curves of the nanoprobes on a Petri dish surface**

**Figure S4**. **F_z_** **curves of nanoprobes on a Petri dish**. The nanoprobes used on the experiments displayed on the Figure 3 of the manuscript were used to perform F_z_ curves directly on a Petri dish as a control experiment: a) 400 nm flat, b) 200 nm flat, c) 200 nm sharp, and d) sharp. As depicted on the panels above, the F_Z_ curves present the expected behavior, showing zero deflection until the nanoprobe reaches de surface, where the vertical force sharply starts to increase. Thus, we can discard artefacts on the F_z_ curves of the experiments due to the small diameter of the used nanoprobes.
